# Value of preoperative spirometry test in predicting postoperative pulmonary complications in high-risk patients after laparoscopic abdominal surgery

**DOI:** 10.1371/journal.pone.0209347

**Published:** 2018-12-19

**Authors:** Tak Kyu Oh, In Sun Park, Eunjeong Ji, Hyo-Seok Na

**Affiliations:** 1 Department of Anesthesiology and Pain Medicine, Seoul National University Bundang Hospital, Seongnam, South Korea; 2 Medical Research Collaborating Center, Seoul National University Bundang Hospital, Seongnam, South Korea; Beth Israel Deaconess Medical Center, UNITED STATES

## Abstract

Whether preoperative spirometry in non-thoracic surgery can predict postoperative pulmonary complications (PPCs) is controversial. We investigated whether preoperative spirometry results can predict the occurrence of PPCs in patients who had undergone laparoscopic abdominal surgery. This retrospective observational study analyzed the records of patients who underwent inpatient laparoscopic gastric or colorectal cancer surgery at Seoul National University Bundang Hospital between January 2010 and June 2017. Preoperative spirometry was performed for patients at a high risk of PPCs, such as elderly patients (age >60 years), patients aged <60 years with chronic pulmonary disease, and current smokers. The main outcome was the association between the results of spirometry tests performed within 1 month prior to surgery and the occurrence of PPCs, as determined by multivariable logistic regression analysis. Of the 898 included patients who underwent laparoscopic gastric (372 patients) or colorectal cancer surgery (526 patients), PPC occurred in 117 patients (gastric cancer: 74, colorectal cancer: 43). A 1% greater preoperative forced vital capacity (FVC) was associated with a 2% lower incidence of PPCs after laparoscopic gastric or colorectal cancer surgery (odds ratio: 0.98, 95% confidence interval: 0.97–0.99, *P* = 0.018). However, the preoperative forced expiratory volume in 1 second (FEV1) (%) and FEV1/FVC (%) were not significantly associated with PPCs (*P* = 0.059 and *P* = 0.147, respectively). In conclusion, lower preoperative spirometry FVC, but not FEV1 or FEV1/FVC, may predict PPCs in high-risk patients undergoing laparoscopic abdominal surgery.

## Introduction

Postoperative pulmonary complications (PPCs) remain an important issue after major surgery performed under general anesthesia, especially in elderly patients or patients with lung diseases. Laparoscopic surgery has many advantages over laparotomy, one of which is the decreased incidence of PPCs [1]. However, PPCs remain important in terms of perioperative management of laparoscopic surgery patients [2]. Atelectasis and subsequent pneumonia can occur after insufflation of high-pressure carbon dioxide into the abdominal cavity after laparoscopic surgeries [3, 4].

Spirometry is a universal, simple, and non-invasive pulmonary function test [5]. Spirometry, along with calculation of the forced expiratory volume in 1 second (FEV1) and forced vital capacity (FVC), is helpful for diagnosing obstructive or restrictive ventilatory defects. These defects are closely related to preoperative chronic obstructive pulmonary disease (COPD), asthma, or interstitial lung disease [6]. Therefore, preoperative spirometry results are used for predicting the occurrence of PPCs, especially in thoracic surgery patients [7]; however, their predictive value in non-thoracic surgery patients has not yet been established. In addition, according to the American College of Physicians guidelines, preoperative spirometry is recommended only in high-risk patients, such as those with COPD or asthma [8].

Given the increased use of laparoscopic surgery, it is important to consider the validity of the American College of Physicians guidelines and the necessity for preoperative spirometry in laparoscopic abdominal surgery patients [8]. Therefore, this study was undertaken to investigate the association between preoperative spirometry results and the incidence of PPCs in patients at a high risk of these complications who underwent laparoscopic abdominal surgery for cancer. We hypothesized that abnormal preoperative spirometry results would be associated with increased occurrence of PPCs after laparoscopic abdominal surgery in such high-risk patients.

## Materials and methods

This retrospective cohort study was conducted with the approval of the institutional review board of Seoul National University Bundang Hospital (SNUBH) (Approval number: B-1710/424-107, Approval date: September 26, 2017). The need for obtaining informed patient consent was waived due to the retrospective nature of the study.

### Patient selection

Medical records of patients who had been diagnosed with gastric or colorectal cancer between January 1, 2010, and June 30, 2017, at SNUBH, and who had undergone laparoscopic surgery under general anesthesia with curative intent, were analyzed. Only those who underwent preoperative spirometry were included in the analysis. The exclusion criteria were as follows: bariatric surgery, intraoperative conversion from a laparoscopic to a laparotomy procedure, incomplete medical records, additional combined resection of extra-abdominal organs, or reoperation during the hospitalization period. All medical records were processed by a medical record technician team, all of whom were blinded to the purpose of the study.

### Laparoscopic colorectal or gastric cancer surgery

Experienced surgical teams performed laparoscopy-guided gastric [9] or colorectal cancer surgery [10]. To improve the surgeon’s intraoperative field of view, a reverse Trendelenburg position was used for laparoscopic gastric surgery and a Trendelenburg position was used for laparoscopic colorectal cancer surgery. Using carbon dioxide insufflation, intraoperative intra-abdominal pressure was maintained between 10 and 20 mmHg, in accordance with the judgment of surgeons and anesthesiologists. All surgeries were performed under general anesthesia and patients were ventilated under the pressure- or volume-controlled mode with a positive end-expiratory pressure. Opioid-based, intravenous, patient-controlled analgesia was used for postoperative pain control; incentive spirometry was applied and actively encouraged by medical staff as postoperative lung care for all patients.

### Spirometry

Physicians conducted preoperative spirometry on the following patient groups: 1) elderly patients aged > 60-years old, 2) patients < 60-years old with a history of preoperative chronic pulmonary disease (e.g., asthma, COPD, or lung cancer), 3) patients < 60-years old who were current smokers. Spirometry tests were performed by a specialized technician from the Department of Pulmonology within 1 month prior to surgery. If the results of the spirometry tests were abnormal, the patient was referred to a pulmonologist and was treated perioperatively as needed. FEV1 (%), FVC (%), and FEV_1_/FVC (%), standardized according to the American Thoracic Society and the European Respiratory Society guidelines, were used to analyze the results of spirometry tests [11].

### PPC diagnosis

In this study, a PPC was defined as a condition involving newly developed pulmonological symptoms, requiring medical or interventional treatment. Pre- and postoperatively, all official radiological findings on chest X-rays and chest computed tomography (CT), white blood cell counts (WBC), C-reactive protein (CRP) levels, and postoperative body temperature were used for the determination of PPC.

The following findings were considered to indicate PPCs: 1) newly developed pleural effusion that had been absent before surgery, which required medical therapy or percutaneous chest tube insertion; 2) symptomatic atelectasis, defined by atelectasis in chest X-ray + oxyhemoglobin saturation < 90% + dyspnea; 3) non-cardiac origin pulmonary edema; 4) postoperative pneumonia; 5) emphysema of the lung (excluding subcutaneous emphysema); or 6) rapid progression of pulmonary complications leading to endotracheal intubation in the intensive care unit. Postoperative pneumonia was defined as an official radiological finding of pneumonia on a chest CT or chest X-ray, as interpreted by a radiologist, accompanied by elevated CRP and WBC or a fever of ≥ 38°C. Two anesthesiologists determined the development of PPCs, in consultation with a pulmonologist; when they were in agreement with each other, the patient was recorded as having PPCs.

### Measurements

The patient characteristics (sex, age, and body mass index) and comorbidities at surgery, including American Society of Anesthesiologists classification, Charlson comorbidity index, history of asthma and chronic obstructive lung disease, and operative characteristics (surgery time [min], intraoperative total fluid input [ml], intraoperative packed red blood cell transfusion, and intraoperative peak airway pressure) were recorded.

### End point

The primary outcome of this study was the association between preoperative spirometry results (FEV_1,_ FVC, and FEV_1_/FVC) and occurrence of PPCs after laparoscopic abdominal surgery.

### Statistical analysis

Student’s *t*-tests and chi-squared tests were used for comparison of continuous and categorical variables, respectively. First, patterns between spirometry results (FEV_1_, FVC, FEV_1_/FVC) and probabilities of PPC occurrence were illustrated by using restricted cubic splines (RCSs). RCSs were first used to determine the linearity of variables, as shown in S1, S2 and S3 Figs. Preoperative FEV1, FVC, and FEV_1_/FVC were included as continuous and independent variables in logistic regression analysis. Next, univariable logistic regression analysis was performed to study simple associations between PPCs (as the dependent variable) and other variables in all patients. Multivariable logistic regression analysis was performed by using factors identified with a *P*-value < 0.1 in univariable analysis. After confirming that variance inflation factors between FEV1, FVC, and FEV1/FVC were > 10, FEV1/FVC were included in another multivariable logistic regression model to avoid multi-collinearity. The validity of the multivariable model was tested using Hosmer–Lemeshow statistics. IBM SPSS version 24.0 was used for all analyses, and R version 3.3.2 was used for cubic spline analysis. Statistical significance was defined as a *P*-value less than 0.05.

## Results

**Fig 1:**
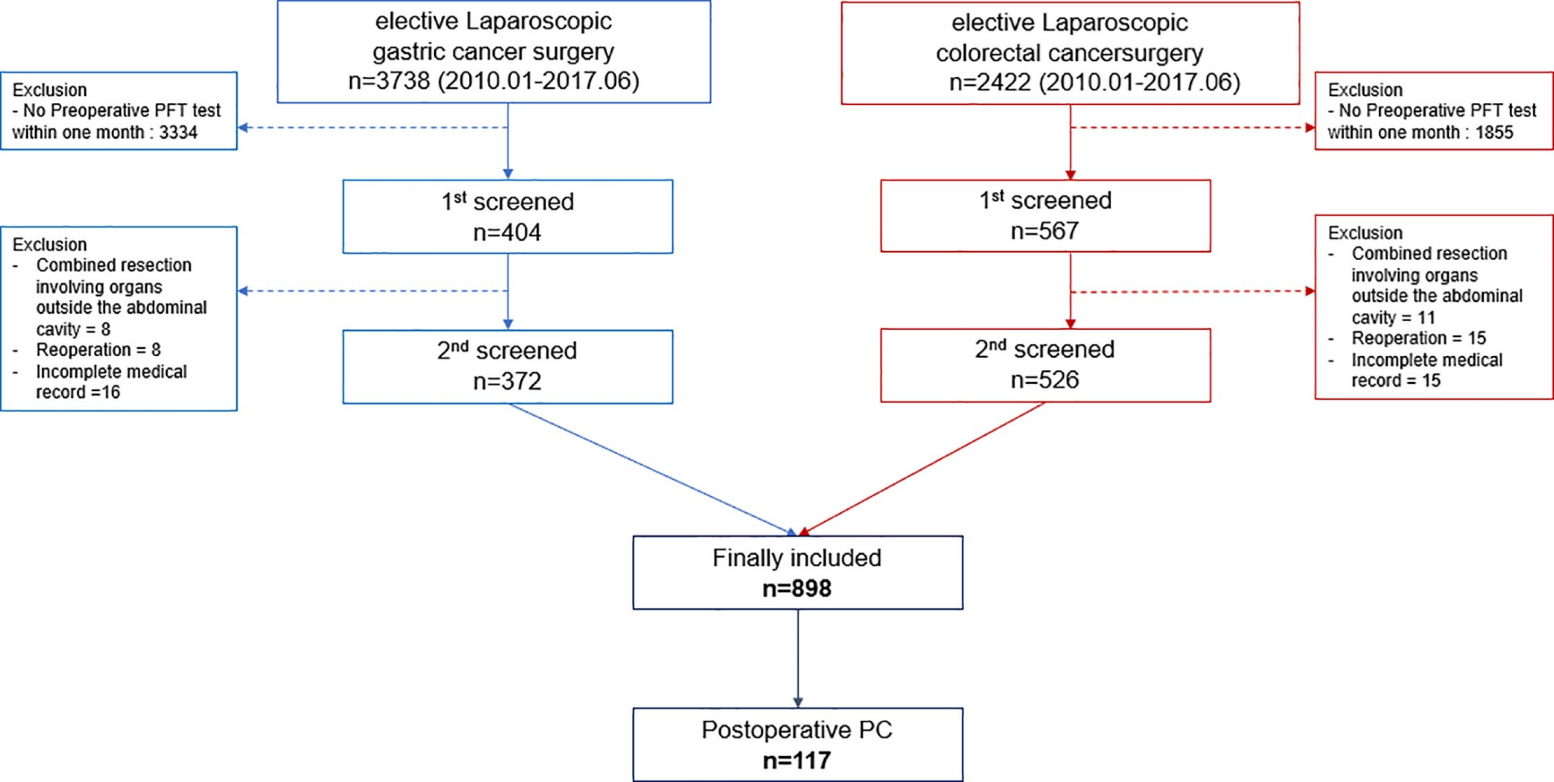
Flow chart of enrollment. PFT, Pulmonary Function Test; PC, Pulmonary Complication.

A total of 6,160 patients underwent laparoscopic gastric or colorectal cancer surgery at SNUBH between January 1, 2010, and June 30, 2017. Of those, 971 patients underwent preoperative spirometry within 1 month prior to surgery. We excluded the following patients: 1) 19 patients were excluded due to combined resection of extra-abdominal organs, 2) 23 patients were excluded due to reoperation during the hospitalization period, 3) 31 patients were excluded due to incomplete medical records. Finally, 898 patients were included in the analyses (Fig 1).

The overall incidence of PPCs was 13.0% (n = 117). Detailed information regarding PPCs is shown in S1 Table. When comparing the clinical characteristics of patients according to the occurrence of PPC, the operation time, anesthesia time, and length of hospital stay were longer in the PPC group than in the no-PPC group (Table 1). Additionally, two patients in the PPC group died during the hospitalization period because of rapid progression of severe pneumonia in the postoperative period.

**Table 1:** Perioperative characteristics of patients with and without postoperative pulmonary complications.

Variables		PPC^a^	No-PPC	*P*-value
		(n = 117)	(n = 781)	
**Age, year**		68.3 (11.4)	65.1 (11.9)	0.006
**Sex: male**		33 (28.2%)	303 (38.8%)	0.027
**Body mass index, kg m**^**-2**^		23.6 (3.6)	23.6 (3.2)	0.895
**ASA class**				0.171
	**1**	25 (21.6%)	234 (30.0%)	
	**2**	80 (69.0%)	479 (61.5%)	
	**≥ 3**	11 (9.5%)	66 (8.5%)	
**Charlson comorbidity index**				0.172
	**0–2**	52 (44.4%)	400 (51.2%)	
	**≥ 3**	65 (55.6%)	381 (48.8%)	
**Type of surgery**				<0.001
	**Gastric cancer**	74 (63.2%)	298 (38.2%)	
	**Colorectal cancer**	43 (36.8%)	438 (61.8%)	
**Preoperative asthma**		3 (2.6%)	18 (2.3%)	0.863
**Preoperative chronic obstructive lung disease**		2 (1.7%)	11 (1.4%)	0.799
**Intraoperative total fluid input, mL**		2,505.8 (1,091.1)	2,304.3 (921.7)	0.033
**Intraoperative packed RBC transfusion**		37 (31.6%)	123 (15.7%)	<0.001
**Intraoperative peak airway pressure**				
	**Mean value, cm H**_**2**_**O**	16.2 (5.2)	16.7 (3.8)	0.179
	**Maximum value, cm H**_**2**_**O**	18.7 (6.1)	19.6 (5.8)	0.120
**Surgery time, min**		199.3 (84.2)	173.3 (64.2)	<0.001
**Anesthesia time, min**		244.8 (86.8)	219.2 (66.7)	<0.001
**Length of hospital stay, day**		17.4 (12.1)	13.5 (6.9)	<0.001
**Postoperative ICU admission**		14 (12.0%)	77 (9.9%)	0.481
**Preoperative FEV1, %**		97.5 (24.4)	104.3 (22.0)	0.002
**Preoperative FVC, %**		94.1 (20.7)	99.9 (16.9)	0.001
**Preoperative FEV1/FVC, %**		71.9 (10.4)	73.4 (9.6)	0.105
**Preoperative DLCO mL mmHg**^**-1**^ **min**^**-1**^		16.9 (3.6)	17.7 (3.9)	0.026

Values are expressed as the mean (standard deviation) or number (%).

^a^PPC, postoperative pulmonary complication; ASA, American Society of Anesthesiologists; ICU, intensive care unit; FEV1, forced expiratory volume in 1 second; FVC, forced vital capacity; DLCO, diffusing capacity of the lungs for carbon monoxide, RBC, red blood cell

### Factors associated with the occurrence of PPCs

The results of univariable logistic regression analysis of covariates for occurrence of PPCs are presented in Table 2.

**Table 2:** Univariable logistic regression analysis of covariates for postoperative pulmonary complications after laparoscopic gastric or colorectal cancer surgery.

Variables				Odds Ratio (95% CI^a^)	*P*–value
**Sex: male**				0.62 (0.40–0.95)	0.028
**Age, year**				1.02 (1.01–1.04)	0.006
**Body mass index, kg m**^**–2**^				0.90 (0.94–1.06)	0.895
**Comorbidities at surgery**					
	**ASA classification**				
			**1**	1	(0.174)
			**2**	1.56 (0.97–2.52)	0.066
			**≥ 3**	1.56 (0.73–3.34)	0.251
	**Charlson comorbidity index**				
			**≥ 3 (vs 0–2)**	1.31 (0.89–1.94)	0.173
	**Asthma**			1.12 (0.32–3.85)	0.863
	**Chronic obstructive pulmonary disease**			1.22 (0.27–5.56)	0.800
**Characteristics of surgery**					
	**Type of surgery**				
		**Colon or rectum**		1	(<0.001)
		**Gastric**		2.79 (1.87–4.17)	<0.001
	**Surgery time, min**			1.01 (1.00–1.01)	<0.001
	**Intraoperative peak airway pressure**				
		**Mean value, cm H**_**2**_**O**		0.97 (0.93–1.01)	0.180
		**Maximum value, cm H**_**2**_**O**		0.97 (0.94–1.01)	0.101
	**Intraoperative total fluid input, L**			1.22 (1.01–1.67)	0.035
	**Intraoperative packed RBC transfusion**			2.47 (1.60–3.82)	<0.001
	**Postoperative ICU admission**			1.24 (0.68–2.28)	0.482
**Year of surgery**					
	**2010–2012**			1	(0.270)
	**2013–2015**			0.90 (0.59–1.36)	0.613
	**2016–2017**			0.60 (0.33–1.11)	0.106
**Preoperative spirometry test**					
	**Preoperative FEV1 (%)**			0.99 (0.98–1.00)	0.002
	**Preoperative FVC (%)**			0.98 (0.97–0.99)	0.001
	**Preoperative FEV1/FVC (%)**			0.98 (0.97–1.00)	0.105

All covariates of *P* < 0.1 in univariable logistic regression analysis were included in multivariable logistic regression analysis.

^a^CI, Confidence interval; ASA, American society of anesthesiologists; RBC, Red blood cell; ICU, Intensive care unit.

Factors identified with *P* < 0.1 in the univariable logistic regression model were selected for inclusion in the final multivariable logistic regression model, and the results of multivariable logistic regression analysis after adjusting for covariates are shown in Table 3. A 1% greater preoperative FVC was associated with a 2% lower PPC incidence after laparoscopic gastric or colorectal surgery (odds ratio [OR]: 0.98, 95% confidence interval [CI]: 0.97–0.99, *P* = 0.018). However, preoperative FEV1 (%) and FEV1/FVC (%) were not significantly associated with PPC (*P* = 0.059 and *P* = 0.147, respectively). In addition, other factors related to the occurrence of PPC after laparoscopic gastric or colorectal surgery were age, type of surgery, duration of surgery, and intraoperative packed red blood cell transfusion, which are already proven as risk factors of PPC [12].

**Table 3:** Multivariable logistic regression analysis of covariates for postoperative pulmonary complications after laparoscopic gastric or colorectal cancer surgery.

Variables				Odds Ratio (95% CI^a^)	*P*–value
**Sex: male**				0.89 (0.55–1.46)	0.653
**Age, years**				1.03 (1.01–1.05)	0.006
**Comorbidities at surgery**					
	**ASA classification**				
			**1**	1	(0.397)
			**2**	1.69 (0.69–4.10)	0.248
			**≥ 3**	1.68 (0.79–3.53)	0.176
**Characteristics of surgery**					
	**Type of surgery**				
		**Colon or rectum**		1	(<0.001)
		**Gastric**		2.90 (1.89–4.46)	<0.001
	**Surgery duration, min**			1.01 (1.00–1.01)	0.003
	**Intraoperative total fluid input, L**			0.98 (0.78–1.24)	0.871
	**Intraoperative packed RBC transfusion**			2.10 (1.30–3.40)	0.002
**Preoperative spirometry test results**					
	**Preoperative FEV1 (%)**^a^			0.99 (0.98–1.01)	0.059
	**Preoperative FVC (%)**^a^			**0.98** (0.97–0.99)	**0.018**
	**Preoperative FEV1/FVC (%)**^b^			1.02 (0.99–1.05)	0.147

All covariates of *P* < 0.1 in univariable logistic regression analysis were included in multivariable logistic regression analysis.

^a,b^included in separate multivariable logistic regression models to avoid multi-collinearity

Hosmer–Lemeshow statistic, chi-squared: 7.752, *P* = 0.458.

CI, Confidence interval; FEV1, forced expiratory volume in 1 second; FVC, forced vital capacity

## Discussion

In this study, we found that a preoperative lower FVC was associated with increased occurrence of PPCs after laparoscopic abdominal surgery, while FEV1 and FEV1/FVC were not. This study is clinically meaningful in that it implicates preoperative spirometry as one of the important factors for predicting PPCs after laparoscopic abdominal surgery in patients at a high risk of these complications, such as elderly patients (aged over 60 years), current smokers, and patients with a history of chronic pulmonary disease.

According to the American College of Physicians guidelines [8], routine preoperative spirometry tests are not recommended for non-thoracic surgery patients, except in cases with preoperative asthma or COPD, although preoperative spirometry has the advantage of identifying previously undiagnosed lung disease [13]. Our institution followed this guideline [8]; thus, preoperative spirometry testing was performed only for elderly patients (aged over 60 years), current smokers, and patients with a history of chronic pulmonary disease, who were relatively more vulnerable to PPCs. The results of this study suggest that ventilation in patients with a lower FVC in these populations should be performed using a more careful strategy (lung protective ventilation) [14] or an optimal fluid administration strategy to reduce the risk of PPCs in the perioperative period [15].

Similar to this study, many previous studies have attempted to evaluate the necessity of preoperative spirometry tests for non-thoracic surgeries. Similar to our study, Tajima et al. found that the FVC (%) results of preoperative spirometry tests were helpful for predicting postoperative pneumonia development in patients who underwent colorectal surgery [16]. In another study, routine preoperative spirometry tests were useful for predicting PPC incidence after bariatric surgery in obese patients [17]. In contrast to these results, Huh et al. reported that preoperative spirometry findings could not be used to stratify the risk of PPC in elderly patients undergoing laparoscopic gastrectomy [18]. These conflicting results may be explained by the fact that PPC is affected by various factors, such as surgery type (laparotomy vs. laparoscopy), surgical site (upper or lower abdomen), intraoperative ventilator care strategy, postoperative lung care strategy, and other patient characteristics [19]. Consequently, a more categorical standard than that provided by the existing guidelines is required in order to assess the necessity for preoperative spirometry, which should be proven through further large population-based studies. In part, the results of our study demonstrated that lower FVC% was related to the PPC-development risk among those patients undergoing laparoscopic abdominal surgery who were at a high risk of these complications preoperatively.

In the present study, the surgical technique was limited to laparoscopic surgery, which has various advantages in terms of post-surgical outcomes, including lung function recovery, over laparotomy [20–22]. However, the intraoperative mechanical ventilation strategy is challenging in laparoscopic surgery, especially when using the Trendelenburg position. During laparoscopic surgery, lung volume and pulmonary compliance are reduced and peak airway pressure is increased. The diaphragm is displaced upward by increased intra-abdominal pressure, which results in a reduction in the functional residual capacity, with a ventilation–perfusion mismatch, and carbon dioxide absorption aggravates hypercapnia [23]. In addition, laparoscopic surgery is more advantageous for postoperative pulmonary function recovery than laparotomy; however, postoperative FVC and FEV1 are decreased even in laparoscopic surgery [24, 25]. From this perspective, it is plausible that preoperative pulmonary function could be a predictive factor in the development of PPCs in patients undergoing laparoscopic abdominal surgery, as demonstrated in this study. Unfortunately, we only found that the lower preoperative FVC was, the higher PPC occurred and could not decide the clear cutoff value of FVC to predict the PPC risk in laparoscopic abdominal surgery. To set the cutoff point of the FVC, an international reference value is required, or a point where the slope changes on a RCS should be identified. However, there was no international reference value of FVC for PPCs and there was also no clear point where the slope changed on RCS of FVC, FEV1, and FEV1/FVC in this study.

This study had a few limitations. First, the generalizability of the study may be compromised, as this was a retrospective single-center study; thus, a prospective multicenter study should be performed in future. Second, we only included patients who underwent laparoscopic gastric or colorectal cancer surgery, and hence bariatric surgery or technically complicated surgeries (e.g., intraoperative conversion from laparoscopic to laparotomy or combined resection of multi-organs) were excluded from this analysis. Lastly, only 898 of 6160 patients underwent preoperative spirometry tests for laparoscopic abdominal surgery in this study, because our center has been performing preoperative spirometry only for elderly patients (aged over 60 years), current smokers, and patients with a history of chronic pulmonary disease, who are relatively more vulnerable to PPCs. Thus, our results may not be generalizable to all patients. However, this study is valuable in that it shows that lower preoperative FVC could be a useful predictor of PPCs after laparoscopic abdominal surgery in patients with a high risk of these complications preoperatively. In this perspective, our findings suggest that more careful perioperative management is needed in this population with lower preoperative FVC.

In conclusion, we found that lower preoperative spirometry FVC might predict development of PPCs in patients undergoing laparoscopic abdominal surgery who are at a high risk of these complications. However, preoperative FEV1 and FEV1/FVC were not associated with the occurrence of PPCs after laparoscopic abdominal surgery for cancer treatment.

## Supporting information

S1 Fig Spline estimates for probability in occurrence of postoperative pulmonary complications, according to preoperative FEV_1_ (%). FEV1, forced expiratory volume in 1 second.
(TIF)

S2 Fig Spline estimates for probability in occurrence of postoperative pulmonary complications, according to preoperative FVC (%). FVC, forced vital capacity.
(TIF)

S3 Fig Spline estimates for probability in occurrence of postoperative pulmonary complications, according to preoperative FEV_1_/FVC (%). FEV_1_, forced expiratory volume in 1 second; FVC, forced vital capacity.
(TIF)

S1 Table Postoperative pulmonary complications. (DOCX)

## References

[1] Schwenk W, Bohm B, Witt C, Junghans T, Grundel K, Muller JM. Pulmonary function following laparoscopic or conventional colorectal resection: a randomized controlled evaluation. Arch Surg. 1999;134(1):6–12; discussion 3. . 9927122 10.1001/archsurg.134.1.6

[2] Joshi GP. Complications of laparoscopy. Anesthesiology Clinics of North America. 2001;19(1):89–105. 11244922 10.1016/s0889-8537(05)70213-3

[3] Sujatha-Bhaskar S, Alizadeh RF, Inaba CS, Koh CY, Jafari MD, Mills SD, et al Respiratory complications after colonic procedures in chronic obstructive pulmonary disease: does laparoscopy offer a benefit? Surg Endosc. 2017 10.1007/s00464-017-5805-5 . 28812150 PMC6281393

[4] Fahy BG, Barnas GM, Flowers JL, Nagle SE, Njoku MJ. The effects of increased abdominal pressure on lung and chest wall mechanics during laparoscopic surgery. Anesth Analg. 1995;81(4):744–50. . 7574004 10.1097/00000539-199510000-00015

[5] Singh SJ, Puhan MA, Andrianopoulos V, Hernandes NA, Mitchell KE, Hill CJ, et al An official systematic review of the European Respiratory Society/American Thoracic Society: measurement properties of field walking tests in chronic respiratory disease. European Respiratory Journal. 2014;44(6):1447–78. 10.1183/09031936.00150414 25359356

[6] Ranu H, Wilde M, Madden B. Pulmonary function tests. Ulster Med J. 2011;80(2):84–90. 22347750 PMC3229853

[7] Dales RE, Dionne G, Leech JA, Lunau M, Schweitzer I. Preoperative prediction of pulmonary complications following thoracic surgery. Chest. 1993;104(1):155–9. . 8325061 10.1378/chest.104.1.155

[8] Qaseem A, Snow V, Fitterman N, Hornbake ER, Lawrence VA, Smetana GW, et al Risk assessment for and strategies to reduce perioperative pulmonary complications for patients undergoing noncardiothoracic surgery: a guideline from the American College of Physicians. Ann Intern Med. 2006;144(8):575–80. . 16618955 10.7326/0003-4819-144-8-200604180-00008

[9] Kim HH, Hyung WJ, Cho GS, Kim MC, Han SU, Kim W, et al Morbidity and mortality of laparoscopic gastrectomy versus open gastrectomy for gastric cancer: an interim report—a phase III multicenter, prospective, randomized Trial (KLASS Trial). Ann Surg. 2010;251(3):417–20. 10.1097/SLA.0b013e3181cc8f6b . 20160637

[10] Kang SB, Park JW, Jeong SY, Nam BH, Choi HS, Kim DW, et al Open versus laparoscopic surgery for mid or low rectal cancer after neoadjuvant chemoradiotherapy (COREAN trial): short-term outcomes of an open-label randomised controlled trial. Lancet Oncol. 2010;11(7):637–45. 10.1016/S1470-2045(10)70131-5 . 20610322

[11] Miller MR, Hankinson J, Brusasco V, Burgos F, Casaburi R, Coates A, et al Standardisation of spirometry. Eur Respir J. 2005;26(2):319–38. 10.1183/09031936.05.00034805 . 16055882

[12] Miskovic A, Lumb AB. Postoperative pulmonary complications. Br J Anaesth. 2017;118(3):317–34. 10.1093/bja/aex002 . 28186222

[13] Watari M, Kumada T, Shimada E, Nagao A. [Preoperative Spirometry Leads Latent COPD Patients to be Discovered and Treated]. Masui. 2015;64(3):331–5. . 26121798

[14] Hemmes SN, Serpa Neto A, Schultz MJ. Intraoperative ventilatory strategies to prevent postoperative pulmonary complications: a meta-analysis. Curr Opin Anaesthesiol. 2013;26(2):126–33. 10.1097/ACO.0b013e32835e1242 . 23385321

[15] Makaryus R, Miller TE, Gan TJ. Current concepts of fluid management in enhanced recovery pathways. Br J Anaesth. 2018;120(2):376–83. 10.1016/j.bja.2017.10.011 . 29406186

[16] Tajima Y, Tsuruta M, Yahagi M, Hasegawa H, Okabayashi K, Shigeta K, et al Is preoperative spirometry a predictive marker for postoperative complications after colorectal cancer surgery? Jpn J Clin Oncol. 2017;47(9):815–9. 10.1093/jjco/hyx082 . 28591816

[17] Clavellina-Gaytan D, Velazquez-Fernandez D, Del-Villar E, Dominguez-Cherit G, Sanchez H, Mosti M, et al Evaluation of spirometric testing as a routine preoperative assessment in patients undergoing bariatric surgery. Obes Surg. 2015;25(3):530–6. 10.1007/s11695-014-1420-x . 25240391

[18] Huh J, Sohn TS, Kim JK, Yoo YK, Kim DK. Is routine preoperative spirometry necessary in elderly patients undergoing laparoscopy-assisted gastrectomy? J Int Med Res. 2013;41(4):1301–9. 10.1177/0300060513489470 . 23908552

[19] Rock P, Rich PB. Postoperative pulmonary complications. Current Opinion in Anesthesiology. 2003;16(2):123–31. 17021450 10.1097/00001503-200304000-00004

[20] Frazee RC, Roberts JW, Okeson GC, Symmonds RE, Snyder SK, Hendricks JC, et al Open versus laparoscopic cholecystectomy. A comparison of postoperative pulmonary function. Ann Surg. 1991;213(6):651–3; discussion 3–4. 1828139 10.1097/00000658-199106000-00016 PMC1358597

[21] Kobayashi T, Nagata H, Kadosaki M, Ohtsuka K. [Changes in cardio-pulmonary function during laparoscopic colectomy and postoperative quality of life—comparison with laparotomy]. Masui. 2006;55(5):579–89. . 16715912

[22] Oshikiri T, Yasuda T, Kawasaki K, Harada H, Oyama M, Hasegawa H, et al Hand-assisted laparoscopic surgery (HALS) is associated with less-restrictive ventilatory impairment and less risk for pulmonary complication than open laparotomy in thoracoscopic esophagectomy. Surgery. 2016;159(2):459–66. 10.1016/j.surg.2015.07.026 . 26361833

[23] Khetarpal R, Bali K, Chatrath V, Bansal D. Anesthetic considerations in the patients of chronic obstructive pulmonary disease undergoing laparoscopic surgeries. Anesth Essays Res. 2016;10(1):7–12. 10.4103/0259-1162.165500 26957682 PMC4767086

[24] Hasukic S, Mesic D, Dizdarevic E, Keser D, Hadziselimovic S, Bazardzanovic M. Pulmonary function after laparoscopic and open cholecystectomy. Surg Endosc. 2002;16(1):163–5. 10.1007/s00464-001-0060-0 . 11961630

[25] Miller TE, Raghunathan K, Gan TJ. State-of-the-art fluid management in the operating room. Best Pract Res Clin Anaesthesiol. 2014;28(3):261–73. 10.1016/j.bpa.2014.07.003 . 25208961

