# Supplementary figures and images for: Value of preoperative spirometry test in predicting postoperative pulmonary complications in high-risk patients after laparoscopic abdominal surgery
Source: PLoS One. 2018 Dec 19;13(12):e0209347. doi: 10.1371/journal.pone.0209347 (PMC6300335; doi:10.1371/journal.pone.0209347)

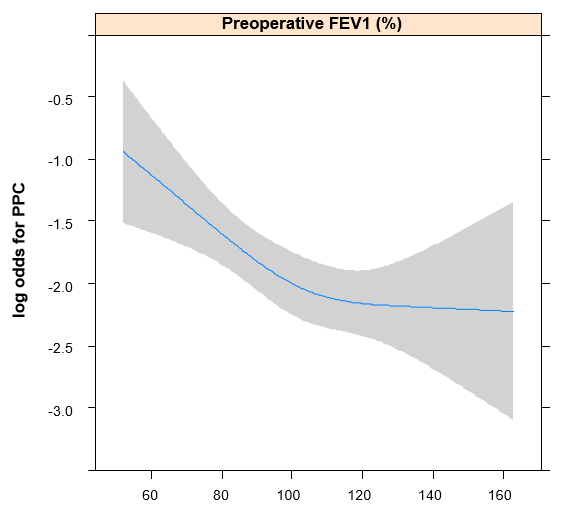

Supplement: S1 Fig — FEV1, forced expiratory volume in 1 second. (TIF) [file pone.0209347.s001.TIF]

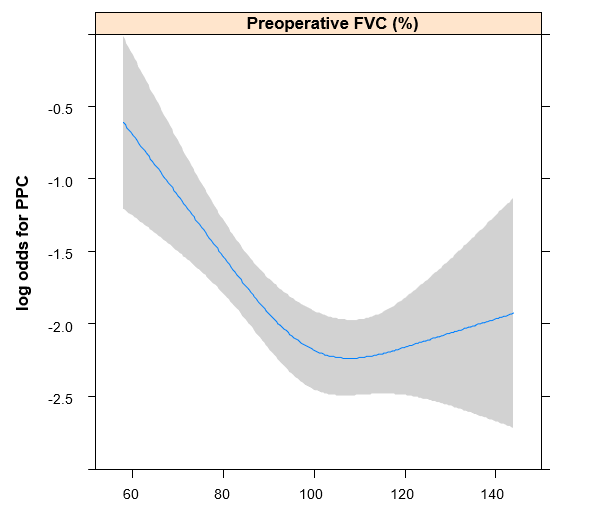

Supplement: S2 Fig — FVC, forced vital capacity. (TIF) [file pone.0209347.s002.TIF]

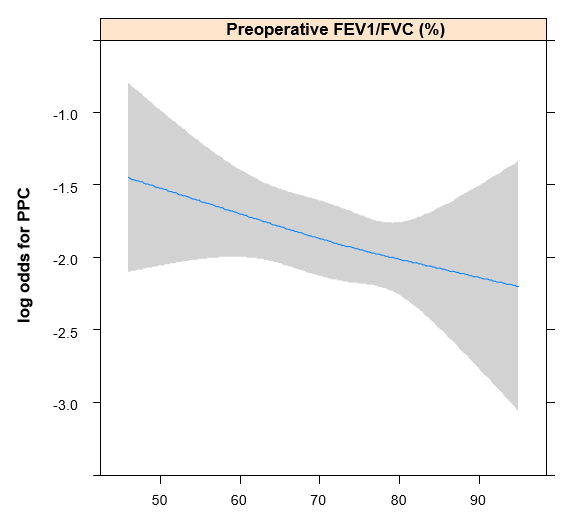

Supplement: S3 Fig — FEV1, forced expiratory volume in 1 second; FVC, forced vital capacity. (TIF) [file pone.0209347.s003.TIF]
