# Supplementary material for: Value of preoperative spirometry test in predicting postoperative pulmonary complications in high-risk patients after laparoscopic abdominal surgery
Source: PLoS One. 2018 Dec 19;13(12):e0209347. doi: 10.1371/journal.pone.0209347 (PMC6300335; doi:10.1371/journal.pone.0209347)
Supplement: S1 Table — (DOCX) [file pone.0209347.s004.docx]

Table S1. Information about postoperative pulmonary complication

|  | Number* | Percent |
| --- | --- | --- |
| Pleural effusion without PCD insertion | 55 | 42.6% |
| Atelectasis with hypoxemia (90%>) and fever (38.0^o^C ≤) | 36 | 27.9% |
| Pneumonia | 15 | 11.6% |
| Pleural effusion with PCD insertion | 14 | 10.9% |
| Emphysema of lung | 7 | 5.4% |
| ARDS with endotracheal intubation | 2 | 1.6% |

*There were 129 pulmonary complication cases for 117 patients

PCD, Percuatneous Chest tube Drainage; ARDS, Acute Respiratory Distress Syndrome
